# Achieving Steroid‐Sparing Remission in Refractory Pyoderma Gangrenosum With an Integrative Meta‐Therapeutic System Based on Traditionally Engineered Nanomercury Sulfide: A Case Series

**DOI:** 10.1155/crdm/9959087

**Published:** 2026-04-29

**Authors:** Magesh Kirubakaran J. P., Pooja Shrivastav, Sangeetha R., Surya Kumar K., Ayumi Saito, Sakthivel T.

**Affiliations:** ^1^ Department of Shalya Tantra, Mahatma Gandhi Ayurveda College & Research Centre, Datta Meghe Institute of Higher Education & Research (Deemed to be University), Salod (H), Wardha, India; ^2^ Department of Shalya Tantra, Sri Jayendra Saraswathi Ayurveda College and Hospital, Nazarathpettai, India; ^3^ Department of Ayurveda, Sri Chandrasekharendra Saraswathi Viswa Mahavidyalaya, Kanchipuram, India; ^4^ Department of Shalakya Tantra, Government Ayurveda Medical College & Hospital, Kottar, Nagercoil, India; ^5^ The Tamil Nadu Dr. M.G.R. Medical University, Chennai, India, tnmgrmu.ac.in; ^6^ Department of Epidemiology, University of Washington, Seattle, Washington, USA, washington.edu

**Keywords:** integrative medicine, Linga Chenduram, mercury sulfide, nanomedicine, pyoderma gangrenosum, refractory disease, steroid-sparing regimen

## Abstract

The management of pyoderma gangrenosum (PG) remains a formidable clinical challenge due to its complex, multipathway immunopathogenesis. Although conventional steroid therapy and advanced biologic agents have improved outcomes, a substantial proportion of patients, particularly those with severe, progressive ulceration, exhibit treatment‐refractory disease, highlighting a critical unmet need for novel strategies capable of network‐level immune modulation. To address this, we designed and implemented a self‐regulating, integrative meta‐therapeutic system centered on a nanostructured, herbo‐metallic core of traditionally processed mercury sulfide (HgS) called Linga Chenduram, a compound characterized by its stable crystalline lattice and a toxicokinetic profile favoring particulate clearance. This system synergistically combines the nanocore with adjunctive phytocompounds (*Allium sativum* and *Aloe vera*), a transient low‐dose glucocorticoid pulse, and an optimized anti‐inflammatory, antioxidant‐rich physiological milieu delivered within a stress‐free environment. In a cohort of four patients with PG refractory to conventional immunosuppressants, this 30‐day protocol elicited a rapid and profound clinical response. Objective metrics demonstrated a mean ulcer area reduction exceeding 61% by Day 30, progressing to complete and sustained re‐epithelialization in all subjects. This robust cutaneous healing occurred concurrently with the normalization of systemic inflammation (C‐reactive protein) and a significant restoration of functional capacity, evidenced by a mean improvement of 35 points on the Anterior Knee Pain Scale. Critically, clinical remission was maintained throughout a 6‐month surveillance period, establishing a definitive steroid‐sparing effect. We propose this multisystem improvements emerge from rational polypharmacology, wherein the nanoengineered HgS core serves as a pivotal immunomodulatory node, potentially engaging master regulatory pathways like NF‐κB to disrupt dysregulated cytokine circuitry. It transcends conventional combination therapy, introducing a paradigm for managing refractory autoinflammation through integrative systems and compelling a rigorous reappraisal of pharmaceutically refined traditional nanomedicines. This preliminary evidence establishes a compelling rationale for future research to deconstruct the system’s architecture and validate its therapeutic potential.

## 1. Introduction

Pyoderma gangrenosum (PG) represents a formidable therapeutic challenge as a recalcitrant autoinflammatory neutrophilic dermatosis characterized by profound multilayered immune dysregulation, where the intricate interplay between innate and adaptive immunity undergoes catastrophic disintegration, driven by a self‐amplifying cytokine storm involving pivotal mediators including interleukin‐1 beta (IL‐1β), interleukin‐6 (IL‐6), the potent neutrophil chemokine interleukin‐8 (IL‐8), interleukin‐17 (IL‐17), interleukin‐23 (IL‐23), and tumor necrosis factor‐alpha (TNF‐α), propagating through hyperactivated Janus kinase (JAK)/signal transducer and activator of transcription (JAK‐STAT) and nuclear factor kappa‐light‐chain‐enhancer of activated B (NF‐κB) cells signaling pathways, with this complex pathophysiological landscape further complicated by genetic predispositions involving mutations in genes such as *PTPN6* and *PSTPIP1* that exacerbate inflammasome dysfunction and immune pathway dysregulation [1–3]. This pathophysiological complexity presents a fundamental translational challenge: The absence of broadly accepted universal treatment guidelines for PG is a direct corollary of the persistent and critical obscurity surrounding its core immunomechanistic dynamics [2]. The documented clinical inadequacy of first‐line systemic corticosteroids and cyclosporine—evidenced by the landmark STOP GAP trial revealing less than 50% healing after six weeks alongside substantial treatment—limiting adverse effects—creates an urgent imperative for innovative steroid‐sparing combination strategies [4], with conventional immunomodulatory agents such as mycophenolate mofetil, methotrexate, dapsone, and minocycline serving as foundational approaches [3, 5], while the contemporary therapeutic arsenal has dramatically expanded to include targeted monoclonal antibodies against TNF‐α (infliximab and adalimumab), IL‐1 (anakinra, canakinumab), IL‐12/23 (ustekinumab), IL‐17/IL‐17 receptor (secukinumab, ixekizumab, brodalumab), and IL‐23p19 (guselkumab, tildrakizumab, risankizumab), alongside small molecule JAK inhibitors (tofacitinib, baricitinib, upadacitinib) [5, 6]; however, the predominantly singular pathway targeting of these advanced biologics may prove insufficient against PG’s concurrently dysregulated multicytokine network, highlighting the critical need for next‐generation meta‐therapeutics capable of simultaneous multipathway immunomodulation, precisely where nanomedicine emerges as a transformative frontier [3–8]. We therefore propose a paradigm‐shifting therapeutic strategy that integrates low‐dose prednisolone with a rationally architected herbo‐metallic nanoconstruct derived from the Siddha formulation Linga Chenduram (LC), featuring a nanostructured mercury sulfide (HgS) core engineered through centuries‐old processing protocols specifically designed to mitigate innate toxicity and enhance biocompatibility [9] and strategically functionalized with *Allium sativum* and *A. vera* to create a bioresponsive, self‐regulating therapeutic vehicle engineered for sophisticated immune modulation. We hypothesize that this integrated complex establishes a novel therapeutic paradigm, functioning as a dynamic polypharmacological system wherein the phytochemical ensemble actively modulates the nanometal–biointerface, governing the supramolecular architecture and release kinetics to achieve orchestrated multitarget engagement with the dysregulated inflammatory network. This system is proposed to operate through emergent self‐regulating behavior, providing sustained immunomodulation while fundamentally serving as a potent steroid‐sparing modality, thereby addressing the critical unmet need in PG through a sophisticated integration of refined traditional nanomedicine within a contemporary mechanistic framework.

## 2. Case Presentation

### 2.1. Case 1: Refractory PG With Seronegative Inflammatory Arthropathy

#### 2.1.1. Clinical Synopsis

A 56‐year‐old male with well‐controlled Type 2 diabetes mellitus (body weight: 89 kg; BMI: 28.7 kg/m^2^) presents with a complex six‐month history of progressive neutrophilic dermatosis. The condition is characterized by a 5 × 7 cm cutaneous ulcer located on the right lower limb superior to the lateral malleolus, accompanied by debilitating bilateral knee arthralgia, more severe on the right side, demonstrating classic features of treatment‐refractory PG.

#### 2.1.2. Diagnostic Odyssey

Initial laboratory investigation 6 months prior to presentation revealed profound systemic inflammation with C‐reactive protein (CRP) 48 mg/L and erythrocyte sedimentation rate (ESR) 58 mm/hr, while comprehensive serological profiling systematically excluded competing diagnoses. Hepatic and renal function panels, including comprehensive liver function tests (LFTs) and kidney function tests (KFTs), were within normal limits. Rheumatoid factor (RF), anticyclic citrullinated peptide (anti‐CCP) antibodies, and antinuclear antibody (ANA) screening returned negative results. Plain radiography of both knees revealed no evidence of degenerative osteoarthritis or erosive changes. Repeated deep tissue cultures maintained sterility throughout incubation, and vascular Doppler studies excluded underlying circulatory pathology.

#### 2.1.3. Pathognomonic Histopathology

**FIGURE 1:**
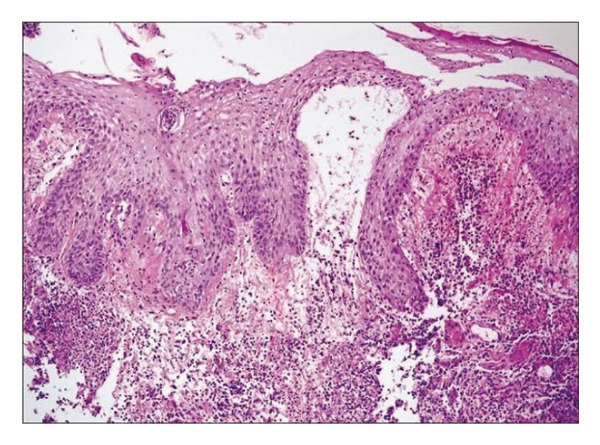
Histopathology feature of Case 1. Photomicrograph (H&E, × 100) of a biopsy from an ulcer edge demonstrating a classic neutrophilic dermatosis. Key features include epidermal ulceration, dense dermal neutrophilic infiltrates with leukocytoclasis (karyorrhectic debris), and marked stromal edema with fibrin deposition. The absence of true vasculitis, granulomas, or micro‐organisms supports the diagnosis of a sterile inflammatory process.

Definitive diagnosis was established through histopathological examination demonstrating characteristic features of neutrophilic dermatosis: dense dermal neutrophilic infiltration with prominent leukocytoclasis, stromal edema, and fibrin deposition in the absence of vasculitis or infection (Figure 1). Application of validated diagnostic instruments yielded unequivocal results: Delphi consensus criteria score of 5 and PARACELSUS score of 16, confirming PG with high specificity and its association with seronegative monoarthropathy.

#### 2.1.4. Therapeutic Trajectory and Disease Chronology

Initial management, started three months prior to the current intervention, involved high‐dose oral prednisolone (escalated from 0.75 to 1 mg/kg/day followed by a six‐week taper), along with topical clobetasol 0.05% and intermittent naproxen 500 mg twice daily. This regimen resulted in only transient therapeutic benefit, with moderate improvement during treatment; however, symptoms recurred upon tapering the corticosteroid. The regimen induced partial ulcer re‐epithelialization and arthralgia mitigation, yet failed to achieve sustained remission. Characteristic of refractory PG, disease activity resurged dramatically upon corticosteroid taper, manifesting as recrudescent ulcer progression and return of debilitating arthralgia.

#### 2.1.5. Current Clinical Presentation

**FIGURE 2:**
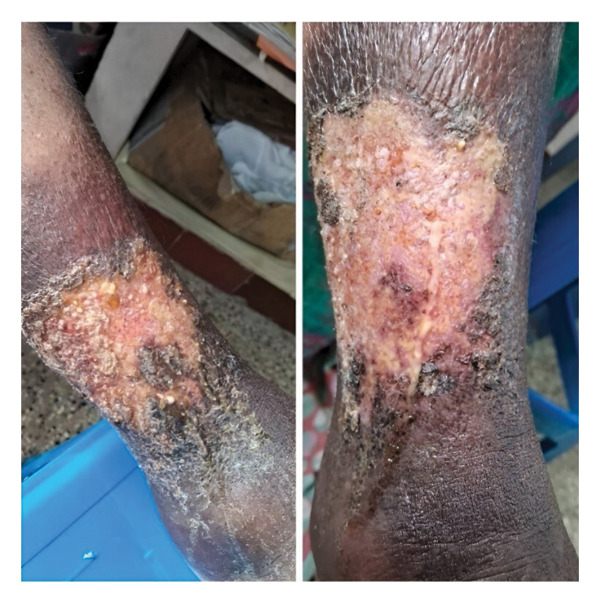
Baseline clinical presentation of refractory pyoderma gangrenosum in Case 1 prior to the integrated intervention. Two views show the ulcer on the right lower limb superior to the lateral malleolus. The lesion exhibits the classic features of a PG ulcer in a state of inflammatory persistence: an irregular, elevated, violaceous, and undermined border with surrounding erythema and cribriform scarring. The superficially clean wound bed belies the underlying pathology, as deep palpation at the time of examination revealed a deep‐seated inflammatory focus with significant tenderness and the expression of thick, purulent exudate.

The present examination reveals the ulcer in a state of suspended chronicity—neither actively expanding nor progressing toward resolution. The lesion persists as a 5 × 7 cm wound with distinctive PG features: elevated violaceous undermined borders maintaining their irregular architecture and a superficially clean wound bed. However, deep palpation reveals a localized area of significant fluctuance and point tenderness, with expression of a thick, purulent exudate, indicating a deep‐seated inflammatory focus not apparent on visual inspection alone (Figure 2). Surrounding cribriform scarring is indicative of previous destructive inflammation (Figure 2). The articular component remains equally problematic, with persistent bilateral knee tenderness (right side greater than left) and functional impairment reflected in an Anterior Knee Pain Scale (AKPS) score of 33/100. Laboratory investigation shows a CRP of 13.6 mg/L. This clinical picture represents the quintessential therapeutic dilemma in PG management: a disease state stabilized enough to avoid hospitalization yet refractory to conventional immunosuppressive strategies, trapped in a cycle of inflammatory persistence without progression to healing.

### 2.2. Case 2: Post‐Traumatic Refractory PG With Seronegative Arthropathy

#### 2.2.1. Clinical Synopsis

A 48‐year‐old male (body weight: 78 kg; BMI: 25.1 kg/m^2^) presents with a five‐month history of a nonhealing, recurrent circular sore on the posterior left ankle and inflammatory bilateral knee arthralgia (left greater than right) with tenderness over left knee following minor trauma, demonstrating the classic pathergy phenomenon and characteristic of severe PG.

#### 2.2.2. Diagnostic Investigation

Laboratory evaluation 5 months prior revealed significant inflammatory markers (CRP 32 mg/L, ESR 52 mm/hr). Comprehensive metabolic profiling revealed normal hepatic and renal function, with all LFT and KFT parameters falling within standard reference ranges. Serological profiling (RF, anti‐CCP, ANA) was negative. Plain radiography of both knees showed no evidence of degenerative or osteoarthritic changes. Repeated sterile deep tissue cultures and vascular Doppler studies excluded infection and circulatory pathology.

#### 2.2.3. Pathognomonic Histopathology

**FIGURE 3:**
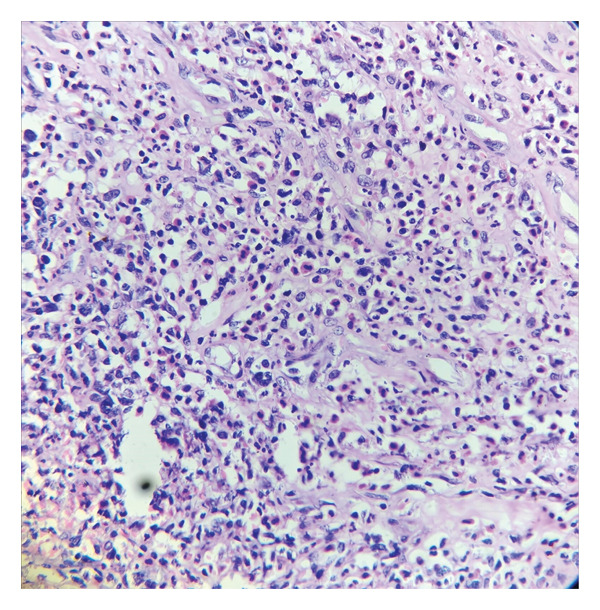
Dense neutrophilic infiltrate observed in Case 2. Photomicrograph (H&E) revealing a dense sheet‐like dermal infiltrate of neutrophils with prominent leukocytoclasis. Early fibrinoid vascular injury and erythrocyte extravasation are present, indicative of incipient neutrophilic vascular damage, without evidence of a primary vasculitis, infection, or granulomatous inflammation suggesting a neutrophilic dermatosis process.

Diagnostic confirmation was established through histopathological examination demonstrating extensive epidermal ulceration with underlying dermal necrosis and dense neutrophilic aggregates throughout superficial and deep dermal layers. Critical findings included perivascular neutrophilic infiltration with early vasculitic changes characterized by fibrinoid necrosis and red blood cell extravasation, in the absence of microbial organisms or granulomatous formation (Figure 3). Application of validated diagnostic instruments yielded definitive scores: Delphi consensus criteria score of 5 and PARACELSUS score of 17, confirming PG associated with seronegative monoarthropathy.

#### 2.2.4. Therapeutic Chronology and Disease Progression

Initial management 5 months prior to the current intervention included conventional wound care with antibiotic therapy showing no clinical response. Subsequent diagnosis led to systemic corticosteroid therapy (prednisolone 1 mg/kg/day) with naproxen 500 mg twice daily, which provided minimal symptomatic relief without meaningful ulcer improvement and reappear after conventional immunosuppression. The disease demonstrated characteristic refractoriness to conventional immunosuppression.

#### 2.2.5. Current Clinical Presentation

**FIGURE 4:**
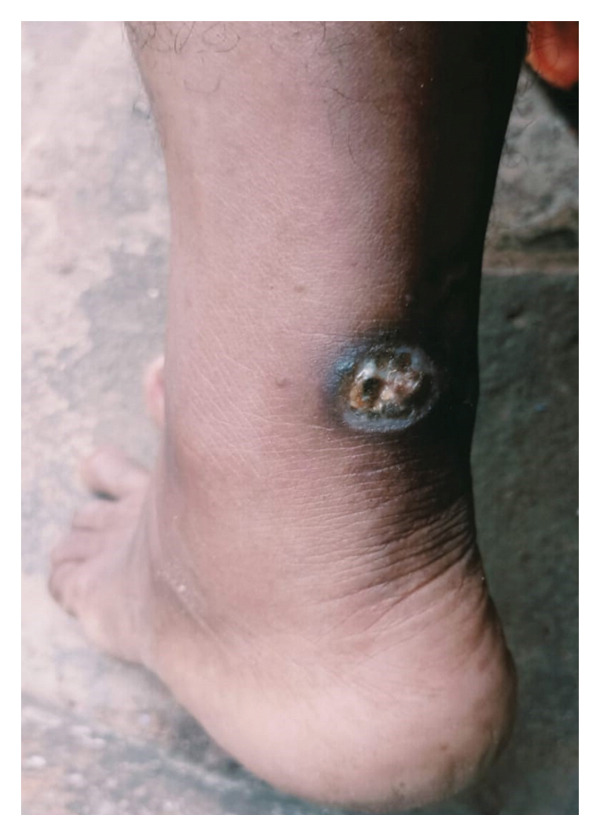
Baseline ulcer of Case 2 prior to integrated intervention. Clinical image depicting a circular, exudative ulcer with an everted, violaceous margin located on the posterior ankle. The lesion was notably tender on palpation.

The present examination reveals a circular ulcer with 1.5 cm radius and 1 cm depth, maintaining classic PG features: elevated everted margins with violaceous hue, clean wound bed with minimal granulation tissue, and surrounding tissue demonstrating chronic inflammatory changes (Figure 4). The lesion is severely tender to palpation with expression of a thin, serous exudate. The articular component remains significantly problematic with persistent bilateral knee pain (left greater than right) reflected in an AKPS score of 54/100 and current CRP of 10.2 mg/L, representing established refractoriness despite appropriate immunosuppressive therapy.

### 2.3. Case 3: Chronic Refractory PG With Treatment‐Resistant Arthropathy

#### 2.3.1. Clinical Synopsis

A 45‐year‐old male with significant comorbidities including heavy smoking (25 pack‐years) and chronic alcohol use disorder (body weight: 63 kg; BMI: 22.1 kg/m^2^) presents with a six‐month progressive history of destructive cutaneous ulceration on the dorsal right foot and debilitating inflammatory arthritis (right greater than left) with marked tenderness and swelling over right knee, representing a complex management scenario of treatment‐refractory PG.

#### 2.3.2. Diagnostic Investigation

Initial laboratory evaluation demonstrated significantly elevated inflammatory markers with CRP 42 mg/L and ESR 62 mm/hr. Comprehensive metabolic profiling revealed normal renal and hepatic function parameters with all LFT and KFT parameters falling within standard reference ranges. Serological studies including RA factor, anti‐CCP, and ANA screening returned negative results. Bilateral knee radiographs revealed no evidence of generative arthropathy, ruling out osteoarthritis, and Doppler scans ruled out vascular pathology. Multiple deep tissue cultures from ulcer margins showed no microbial growth after 48‐h aerobic and anaerobic incubation.

#### 2.3.3. Pathognomonic Histopathology

**FIGURE 5:**
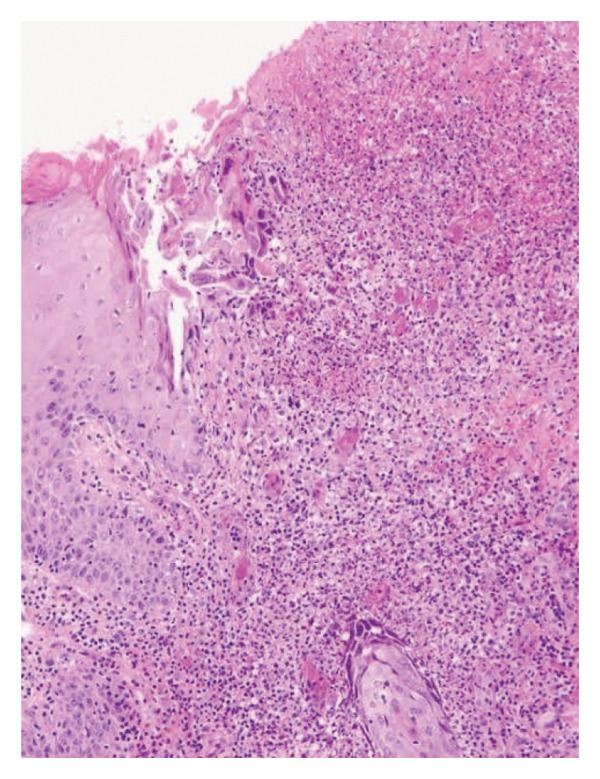
Chronic pyoderma gangrenosum histopathology of Case 3. Photomicrograph (H&E, × 100) showing features of chronicity, including pseudoepitheliomatous hyperplasia at the ulcer margin and well‐formed neutrophilic microabscesses within the dermis. The presence of leukocytoclasis in an edematous stroma, in the absence of vasculitis or infection, confirms the neutrophilic dermatosis.

Diagnostic confirmation was established through histopathological examination demonstrating characteristic features of chronic PG: pseudoepitheliomatous hyperplasia at ulcer borders with extensive dermal neutrophilic microabscess formation and neutrophil‐predominant interstitial inflammation. The specimen showed an intact basement membrane architecture with complete absence of infectious organisms or granulomatous formation (Figure 5), confirming sterile neutrophilic dermatosis. Application of validated diagnostic instruments yielded definitive scores, confirming the diagnosis with high specificity: Delphi consensus criteria score of 5 and a PARACELSUS score of 17 confirm the diagnosis of PG associated with seronegative monoarthropathy.

#### 2.3.4. Therapeutic Chronology and Disease Progression

Initial management 6 months prior to current intervention included a 4‐week course of systemic corticosteroid therapy (prednisolone 1 mg/kg/day) with concurrent naproxen 500 mg twice daily and topical clobetasol 0.05% application. This regimen achieved transient partial improvement with approximately 40% ulcer size reduction and moderate symptomatic relief. However, characteristic disease relapse occurred within 3 months following therapy discontinuation, with recurrence of aggressive ulceration and progressive arthralgia despite initial response.

#### 2.3.5. Current Clinical Presentation

**FIGURE 6:**
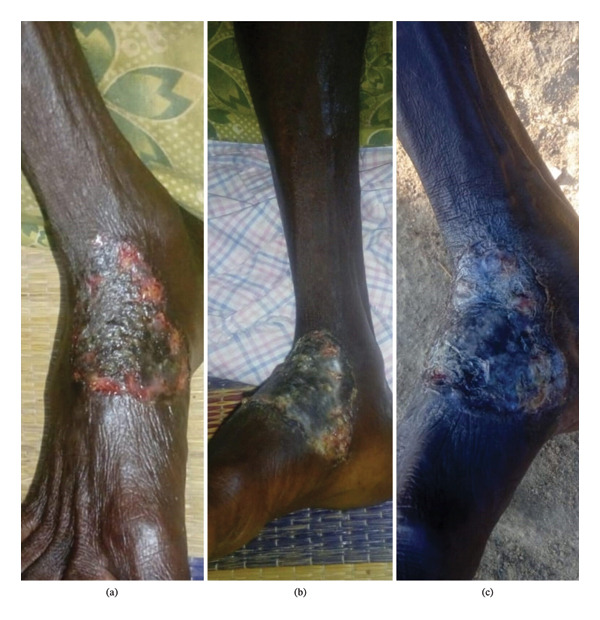
Disease progression in refractory Case 3. Serial clinical photographs documenting the ulcer on the plantar ankle: (a) Status after a prior course of prednisolone; (b) 6 months prior without immunosuppressive therapy; (c) active, purulent ulcer with a progressive margin at baseline, immediately before the current integrated regimen.

Physical examination reveals a 5 × 3 cm purulent ulcer on the right plantar surface with irregular elevated margins and surrounding inflammatory halo and severe tenderness on palpation (Figure 6). The lesion demonstrates classic PG features including undermined violaceous borders, minimal granulation tissue formation, and peripheral cribriform scarring. Concurrent bilateral knee arthralgia persists (right greater than left) with tenderness over the right knee with significant functional limitation reflected in an AKPS score of 39/100 and current CRP of 12.4 mg/L. The clinical presentation confirms disease refractoriness to conventional immunosuppressive therapy, highlighting the need for novel therapeutic approaches in high‐risk patient populations.

### 2.4. Case 4: Persistent PG With Incomplete Therapeutic Response

#### 2.4.1. Clinical Synopsis

A 42‐year‐old female (body weight: 65 kg; BMI: 23.1 kg/m^2^) presents with a four‐month history of characteristic twin ulcerations in the postero‐superior to right calcaneal region accompanied by inflammatory arthralgia (right greater than left) with noticeable swelling and tenderness on palpitation over right knee, demonstrating a pattern of partial steroid responsiveness without achieving complete remission, consistent with persistent PG.

#### 2.4.2. Diagnostic Investigation

Laboratory analysis revealed elevated inflammatory markers with CRP 24 mg/L and ESR 38 mm/hr. Laboratory evaluation of liver and kidney function showed no abnormalities, with LFT and KFT values all within normal limits. Comprehensive serological profiling including RF, anti‐CCP antibodies, and ANA returned within normal parameters. Bilateral knee x rays showed a normal pattern without evidence of degenerative arthropathy, and Doppler studies revealed no significant vascular pathology. Deep tissue cultures from both ulcer sites showed no microbial growth after 48‐h incubation.

#### 2.4.3. Pathognomonic Histopathology

**FIGURE 7:**
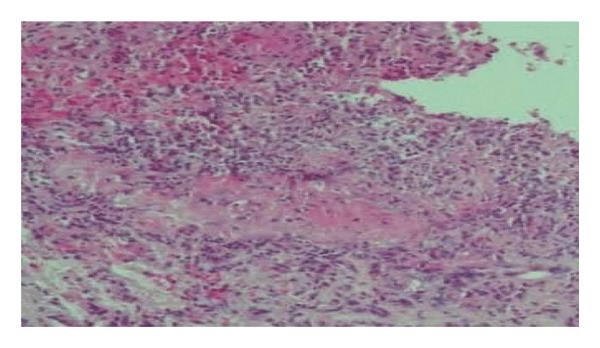
Histopathology of persistent pyoderma gangrenosum. Low‐power view (H&E) showing extensive dermal necrosis with a mixed neutrophilic and lymphoplasmacytic infiltrate, leukocytoclasis, and fibrinous debris, consistent with a severe, persistent sterile neutrophilic dermatosis.

Histopathological examination confirmed the diagnosis, demonstrating dense dermal neutrophilic infiltration with mixed chronic inflammatory cells and characteristic leukocytoclasis. The specimen showed intact vascular architecture without evidence of vasculitis, infection, or granulomatous formation (Figure 7), consistent with sterile neutrophilic dermatosis. The diagnosis was further validated using standardized scoring systems, yielding a Delphi consensus criteria score of 4 and a PARACELSUS score of 10, solidifying the diagnosis of persistent PG associated with seronegative monoarthropathy.

#### 2.4.4. Therapeutic Chronology and Disease Progression

Four months prior to the current intervention, the patient underwent a 4‐week course of systemic corticosteroid therapy (prednisolone 0.75 mg/kg/day) with concurrent ibuprofen 400 mg three times daily, resulting in significant but incomplete clinical improvement. While the regimen produced a substantial reduction in pain and inflammatory signs, complete ulcer resolution was not achieved, and disease activity persisted at a low level throughout therapy.

#### 2.4.5. Current Clinical Presentation

**FIGURE 8:**
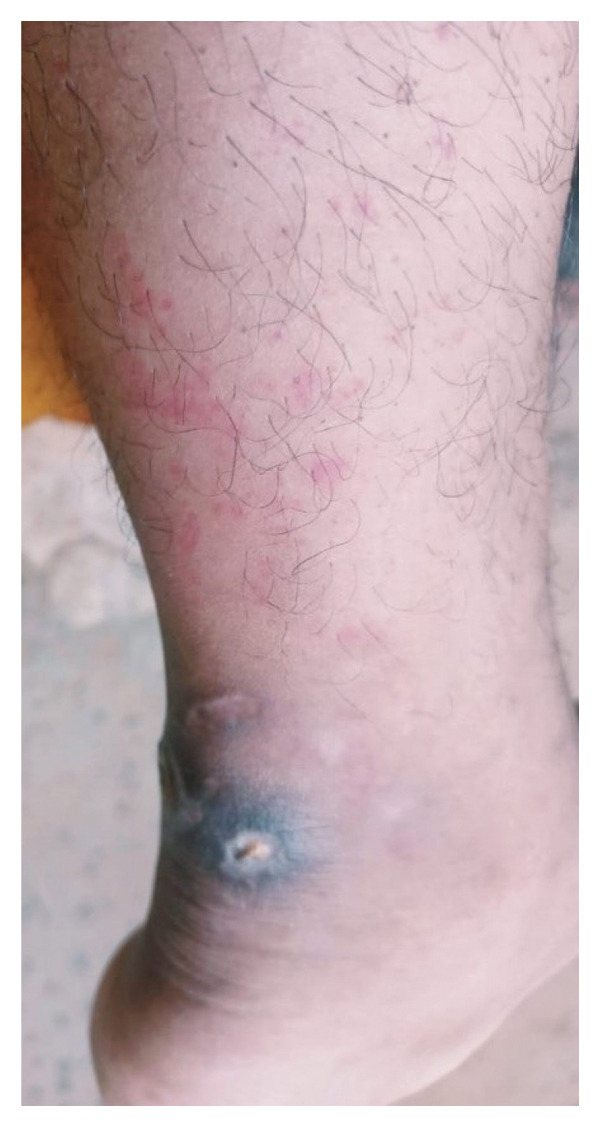
Clinical photograph showing a circular, exudative ulcer over the posterosuperior calcaneal region at the start of the 30‐day integrative treatment protocol. The ulcer was severely tender on palpation.

Physical examination reveals persistent twin circular ulcerations with a combined surface area of 3.1 cm^2^, each measuring < 1 cm radius, located posterior superior to the right calcaneal region. The lesions maintain characteristic PG features with everted violaceous borders, minimal exudate, and surrounding erythema with severe tenderness over palpation (Figure 8). Bilateral knee arthralgia persists (right is greater than left) with noticeable swelling and tenderness over the right knee with functional impact reflected in an AKPS score of 71/100 and current CRP of 8.3 mg/L. This presentation exemplifies the challenging clinical scenario of partially responsive PG, where conventional therapy achieves symptomatic control without inducing complete disease remission.

## 3. Intervention

**FIGURE 9:**
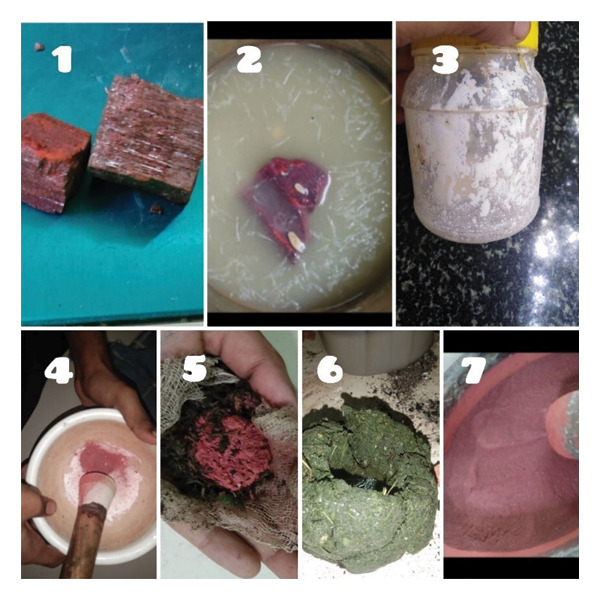
Traditional pharmaceutical preparation of Linga Chenduram. The sequential steps depict the Siddha synthesis and detoxification of nanoengineered HgS: (1) raw HgS; (2) detoxification with lemon juice; (3, 4) trituration with *Euphorbia tortilis* latex; (5) the dried HgS cake coated with a herbal paste of *Pergularia daemia* and *Calotropis procera*; (6) incineration in a sealed ceramic vessel at 300°C; (7) The final product, Linga Chenduram.

All four patients received a 30‐day integrative regimen comprising a 14‐day tapered oral prednisolone course (10 mg/day for 7 days followed by 5 mg/day for 7 days) administered one hour before the herbo‐metallic complex, which consisted of a total daily dose of 200 mg of the Siddha nanoformulation LC [9–11], administered as 100 mg twice daily with 25 mL of a freshly prepared *Al. sativum–A. vera* decoction (standardized from 5 gm garlic scapes/bulbs paste and 10 gm *Aloe vera* pulp reduced from 150 to 25 mL), complemented by topical *A. vera* gel application (Table 1). The LC was prepared through a rigorous, multistage Siddha protocol to ensure detoxification and nanoformulation: Raw HgS was first subjected to seven cycles of immersion and complete desiccation in fresh *Citrus limon* juice, a primary purification step that x‐ray diffraction (XRD) analysis confirmed transformed impure raw HgS into a product with an exceptionally pure trigonal trapezohedral crystalline structure [12]. This purified HgS was then triturated with *Euphorbia tortilis* latex for 12 h, and the resulting dried cake was encapsulated in a fresh herbal paste of *Pergularia daemia* and *Calotropis procera*, sealed in an air‐tight ceramic vessel, and incinerated in a muffle furnace using a controlled temperature protocol (gradually raised to 300°C, maintained, and cooled to room temperature before opening), with this entire incineration cycle repeated seven times to yield the final dark reddish, acid‐insoluble LC (Figure 9) [9, 10]. Comprehensive physicochemical characterization confirmed its nanoscale properties, with scanning electron microscopy (SEM) revealing a primary particle size of 10–100 nm and chemical analysis verifying a defined composition of mercury (33%–35%) and sulfur (6%–7.5%) with a complete absence of free mercury [9, 10]. This entire pharmaceutical protocol was delivered within a strictly controlled environment featuring a low‐sodium diet rich in cucumber, pumpkin, gooseberry, almonds, walnuts, and green leafy vegetables, with strict prohibition of junk, fried, and sour–spicy foods, and was supported by optimized sleep hygiene, a pollution‐free atmosphere, and minimization of all physical and mental stressors to create a holistic, system‐wide therapeutic intervention.

**TABLE 1:** Standardized 30‐day inpatient integrative intervention protocol for refractory pyoderma gangrenosum with seronegative monoarthropathy.

S. no	Drug/intervention	Composition/preparation	Dosage	Frequency	Administration timing	Duration
1.	Linga Chenduram	Nanoformulated cinnabar (HgS) via traditional Siddha processing [9–11]	100 mg	Twice daily (total: 200 mg/day)	With garlic + *Aloe vera* decoction, before food	30 days

2.	Garlic–*Aloe vera* decoction	5 gm fresh garlic scapes + bulbs paste + 10 gm fresh *Aloe vera* pulp boiled in 150 mL water reduced to 25 mL	25 mL	Twice daily	Before food, with LC	30 days

3.	Tablet prednisolone	Synthetic glucocorticoid	10 mg	Once daily	Morning, 1 h before LC	7 days

4.	Tablet prednisolone	Synthetic glucocorticoid	5 mg	Once daily	Morning, 1 h before LC	7 days

5.	*Aloe vera* gel	Fresh *Aloe barbadensis* gel	—	External application	Topical to ulcer sites	30 days

6.	Supportive care	Diet: Ant‐oxidative, phytonutrient‐rich, low‐sodium diet. Prohibited: junk food, fried items, sour–spicy foods. Provided: cucumber, pumpkin, gooseberry, almonds, walnuts, green leafy vegetables. Environment: Pollution‐free accommodation with minimized physical/mental stressors and optimized sleep hygiene.	Supportive Care			.

## 4. Outcome and Result

**FIGURE 10:**
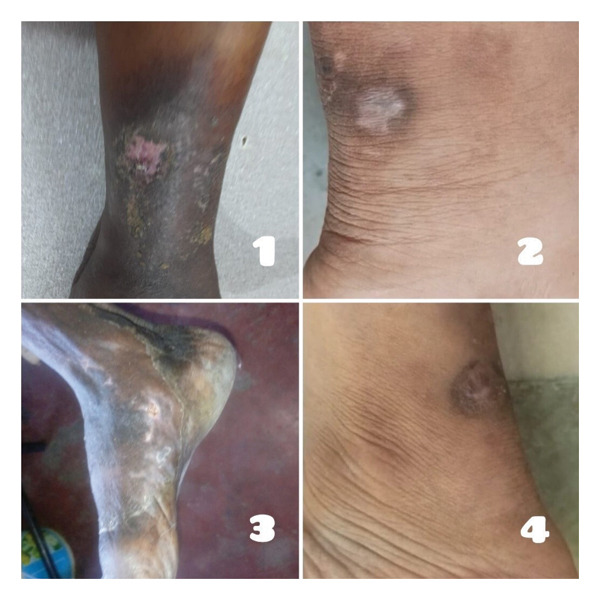
Complete wound closure achieved postintervention in all four cases. Clinical images show the final healed state of refractory PG ulcers across all four cases: (1) Case 1 at 75 days, (2) Case 4 at 40 days, (3) Case 3 at 80 days, (4) Case 2 at 45 days following the initiation of the integrative meta‐therapeutic protocol.

The implementation of the 30‐day integrated therapeutic protocol yielded substantial and multifaceted clinical improvements across all four patients with refractory PG associated with seronegative monoarthropathy (Figure 10). Objective wound metrics confirmed a pronounced and progressive reduction in the ulcer surface area, with mean area reduction exceeding 61% by Day 30 (Table 2). A concomitant marked reduction in ulcer tenderness was noted. This robust cutaneous response was paralleled by a swift and significant amelioration of systemic inflammation, evidenced by a marked decline in CRP levels, which normalized or neared the normal range (< 5 mg/L) in all cases (Table 3). Concomitantly, patients reported a dramatic enhancement in functional mobility and quality of life, quantified by a mean increase of 35 points on the detailed AKPS score, reflecting a resolution of debilitating arthralgia with complete absence of tenderness on palpation (Table 4). Analysis of healing kinetics revealed distinct patient trajectories, delineating rapid responders (Cases 2 and 4, achieving full re‐epithelialization within 40–45 days), a moderate responder (Case 1, ∼75 days), and a delayed yet ultimately successful responder (Case 3, ∼80 days), a heterogeneity consistent with the complex pathophysiology of PG. Critically, during a 6‐month surveillance period, sustained remission was observed with no evidence of elevation in KFT and LFT markers and with no instances of ulcer recurrence, disease flare, or treatment‐emergent adverse events, as rigorously monitored through serial hematological, renal, and hepatic function panels. Comprehensive heavy metal biomonitoring performed at the 6‐month follow‐up revealed serum and urine mercury levels that were negligible and well within standard safety thresholds for environmental exposure (Table 5), yielding direct human toxicokinetic data indicative of efficient systemic clearance. This triad of outcomes—objective wound closure, resolution of systemic inflammation, and functional restoration—collectively underscores the potential of this integrative regimen as a viable strategy for managing recalcitrant PG associated with seronegative monoarthropathy.

**TABLE 2:** Longitudinal assessment of wound healing parameters.

Patient	Initial ulcer area (cm^2^)	Ulcer area at day 30 (cm^2^)	Percentage reduction in area (%)	Initial ulcer depth (mm)	Ulcer depth at day 30 (mm)	Time to complete healing (days)
Case 1	35.0	14.0	60.0	5.0	2.0	∼75
Case 2	7.1	1.0	85.9	10.0	2.0	∼45
Case 3	15.0	7.5	50.0	4.0	2.0	∼80
Case 4	3.1	0.5	83.9	3.0	0.5	∼40

*Note:* This table quantifies the objective cutaneous response to the 30‐day integrative intervention, documenting the progressive reduction in ulcer dimensions.

**TABLE 3:** Systemic inflammatory biomarker response to intervention.

Patient	CRP before treatment (mg/L)	CRP after treatment (mg/L)
Case 1	13.6	6.7
Case 2	10.2	4.8
Case 3	12.4	5.8
Case 4	8.3	4.2

*Note:* Serial measurements of C‐reactive protein levels pre‐ and post‐30‐day meta‐therapeutic protocol. The normalization of CRP values correlates with the observed clinical resolution of inflammation, underscoring the systemic immunomodulatory effect of the treatment.

**TABLE 4:** Detailed Anterior Knee Pain Scale scores before treatment (BT) and after treatment (AT) treatment total score are out of 100.

AKPS parameter	Scoring criteria	Case 1		Case 2		Case 3		Case 4	
		BT	At	BT	At	BT	At	BT	At
1. Limp	None (5), Slight (3), Constant (0)	3	5	3	5	3	5	5	5
2. Support	None (5), Painful (3), Impossible (0)	3	5	3	5	3	5	3	5
3. Walking	Unlimited (5), > 2 km(3), 1–2 km (2), Unable(0)	0	2	2	5	2	5	2	3
4. Stairs	No difficulty (10), Slight Pain (8), Pain (5), Unable (0)	5	8	5	10	5	8	5	8
5. Squatting	No difficulty (5), Repeated Painful (4), Painful (3), Partial Weight (2), Unable (0)	0	2	3	4	2	3	3	4
6. Running	No difficulty (10), Pain > 2 km (8), Slight Pain (6), Severe (3), Unable (0)	3	6	3	8	3	6	6	8
7. Jumping	No Difficulty (10), Slight Difficulty (7), Constant Pain (2), Unable(0)	0	7	0	7	2	7	7	10
8. Prolonged Sitting	No difficulty (10), Pain After Exercise (8), Constant Pain (6), Pain Forces Extension (4), Unable (0)	0	8	8	10	0	4	6	8
9. Pain	None (10), Slight Occasional (8), Interferes Sleep (6), Occasionally Severe (3), Constant Severe (0)	0	8	3	8	3	8	6	8
10. Swelling	None (10), After Exertion (8), After Daily Activities (6), Every Evening (4), Constant (0)	4	10	6	8	6	8	8	10
11. Patellar Motion	None (10), Occasional (4), One Dislocation(2), > 2 Dislocations (0)	10	10	10	10	10	10	10	10
12. Thigh Atrophy	None (5), Slight (3), Severe (0)	5	5	5	5	0	3	5	5
13. Flexion Deficiency	None (5), Slight (3), Severe (0)	0	5	3	5	0	3	5	5
Total Score	100	33	81	54	85	39	62	71	89

*Note:* A higher score indicates better function and less pain.

**TABLE 5:** Serum and urine mercury levels at six‐month follow‐up.

Patient	Serum mercury (μg/L)	Urine mercury (μg/g creatinine)
Case 1	2.00	4.0
Case 2	1.75	3.0
Case 3	3.25	5.0
Case 4	4.75	2.0
Typical safety reference	< 5–10	< 5–20

## 5. Discussion

PG represents a paradigm of immune dysregulation where conventional single‐pathway interventions consistently demonstrate limited efficacy against the disease’s robust inflammatory networks. The concurrent presentation of refractory cutaneous ulceration with seronegative monoarthropathy in our cohort underscores PG’s nature as a systemic autoinflammatory disorder, characterized by pan‐immunological disruption that transcends dermatological manifestations. This clinical complexity is further exemplified by the pathergic response observed in Case 2, where minor trauma may potentially initiate IL‐30–mediated immune activation, revealing a system perpetually primed for exaggerated inflammatory responses [1–5].

The consistent multisystem remission observed across all cases suggests the emergence of a sophisticated bioresponsive, self‐regulating therapeutic architecture. This system, architected around a nanostructured herbo‐metallic core of engineered HgS nanoparticles in the form of LC, appears to operate as a polypharmacological network modulator [9, 10]. By simultaneously engaging innate and adaptive immune pathways, it achieves the coordinated disruption of PG’s self‐sustaining inflammatory circuitry, effectively recalibrating immune homeostasis where single‐pathway agents have struggled to achieve sustained efficacy [4].

Within this therapeutic landscape, our integrative regimen demonstrates compelling clinical evidence of noticeable engagement with shared pathogenic pathways. The simultaneous resolution of both cutaneous and articular inflammation, evidenced by noticeable wound re‐epithelialization, normalized inflammatory markers, and restored functional capacity, indicates the fundamental restoration of immune homeostasis. This transformation of treatment‐refractory wounds toward complete healing, achieved alongside arthropathy resolution, suggests our meta‐therapeutic framework successfully addresses the core network pathology of PG, positioning it as a promising paradigm for managing this complex autoinflammatory disorder.

### 5.1. Reconceptualizing Mercury: A Scientific Reappraisal From Toxin to Precision Therapeutic

The incorporation of a mercury‐based compound in a modern therapeutic protocol necessitates a rigorous scientific reappraisal of one of medicine’s most enduring dichotomies [13]. The well‐documented toxicity of most of the mercury species, such as methylmercury and mercury chloride, is undeniable, with mechanisms involving oxidative stress, proinflammatory cytokine induction, and autoimmune activation [14, 15]. This established toxicological profile rightfully warrants extreme caution [16]. On the other hand, centuries‐old traditional systems such as Siddha, Ayurveda, and traditional Chinese medicine demonstrate a sophisticated, deliberate utilization of processed mercury, documenting both its potent therapeutic applications for complex diseases and its inherent toxic potential [13, 17–19]. This historical context suggests these systems developed advanced pharmaceutical protocols not in ignorance of mercury’s dangers, but precisely to transmute its potent bioreactivity into a controlled therapeutic force [20–23].

The compelling safety profile of the nanostructured HgS in LC is not merely an absence of adverse effects but is demonstrated through a multitiered toxicological validation that meets modern regulatory standards. This safety is rooted in the unique physicochemical properties conferred by traditional pharmaceutical processing, which fundamentally alters the toxicokinetics and toxicodynamics of mercury. Acute and subacute toxicity studies conducted in accordance with OECD guidelines form the first line of evidence, establishing an LD_50_ for LC exceeding 2000 mg/kg and a no‐observed‐adverse‐effect level (NOAEL) of 180 mg/kg/day, with no significant alterations in hematological, serum biochemical, or histopathological parameters [23]. This safety profile is further substantiated over the long term; a landmark 180‐day chronic toxicity study on Rasaraj Rasa—a cognate herbo‐metallic formulation—confirmed a NOAEL at 270 mg/kg (five times the human dose), with no treatment‐related toxicological findings observed in any endpoint, including comprehensive gross and histopathological examinations [24]. The consistency of this finding is reinforced by a 90‐day study on Makaradhwaja, which also identified a clear NOAEL, demonstrating the medium‐term safety of properly processed HgS‐based drugs [25]. The reproducibility of this safety across different Siddha formulations is highlighted by research on Pũrṇa Cantirotaya Centũram, which similarly reported a high LD_50_ and an absence of adverse effects in a 28‐day repeated dose study [26]. Critically, this preclinical evidence translates to a favorable clinical safety profile. In our cohort, serum and urine mercury levels measured at the 6‐month follow‐up were negligible (Table 5), remaining within standard reference ranges for environmental exposure. This indicates a lack of significant bioaccumulation and is consistent with a disposition profile dominated by the efficient renal clearance of the intact, nondissociated nanoarchitecture, rather than the tissue sequestration characteristic of ionic or organic mercury species.

The critical differentiator lies in the physicochemical speciation and nanoarchitectural context [17–21, 27]. The LC used in this study is not elemental or ionic mercury, but a nanoengineered HgS compound produced through a rigorous, multistage Siddha protocol of purification and incineration as detailed in the intervention [9–11]. This process yields a material with distinct properties: a defined trigonal crystalline structure, a primary particle size in the 15–100 nm range, and a composition verified to be free of unbound mercury ions [9, 10]. These characteristics fundamentally alter its toxicokinetics by its stable crystalline structure that minimizes dissociation and the release of bioactive Hg^2+^, thereby enabling its proposed bioactivity to be mediated through controlled nanoparticulate interactions rather than indiscriminate ionic toxicity [18]. While direct quantitative comparisons require careful contextualization, extensive toxicological research consistently confirms that HgS exhibits lower tissue accumulation than methylmercury or mercury chloride, underpinning its historical and potential therapeutic utility [18, 22]. This fundamental physicochemical distinction is quantitatively reflected in the comparative toxicology literature, where cinnabar (α‐HgS) is consistently characterized as orders of magnitude less toxic than methylmercury or mercury chloride [18].

Critically, the mechanistic basis for this documented safety is elucidated by advanced material characterization, which confirms that a traditional synthesis yields a highly stable, crystalline α‐HgS phase. X‐ray absorption fine structure (XAFS) spectroscopy analyses reveal a robust, defect‐free (< 3%) nanocrystalline structure, with a complete absence of free mercury (Hg^0^) or organomercurial compounds—the primary toxicological concern [27]. This specific speciation is the decisive factor. The strong covalent Hg–S bond within the crystalline lattice presents a formidable kinetic barrier to dissociation, preventing the release of bioavailable Hg^2+^ ions and subsequent oxidative stress and protein dysfunction that characterize mercury toxicity. This is conclusively validated in functional biological assays. Studies on Sidh Makardhwaj demonstrate that even at five times the human equivalent dose, the formulation induces no significant neurobehavioral deficits, no alteration in cerebral acetylcholinesterase activity, and no impairment of liver or kidney function, despite a dose‐dependent accumulation of mercury in tissues that remains sequestered in its nonreactive sulfide form [28]. At the cellular level, α‐HgS exhibits no significant cytotoxicity up to 75 ppm in NIH3T3 cells, in stark contrast to the intermediate β‐HgS, which induces toxicity above 20 ppm due to greater cellular uptake, highlighting the critical role of the final crystalline structure and nanomorphology in ensuring biocompatibility [29]. Furthermore, the characteristically low toxicity of the traditionally processed nano‐HgS core is mechanistically substantiated by comparative in vivo studies, which demonstrate that its robust crystalline lattice prevents the dissociation and systemic toxicity that drive the pathology of ionic mercury. Unlike mercury chloride—which induces neurotoxicity in zebrafish via elevated cortisol and locomotor deficits, and nephrotoxicity in mice through significant renal accumulation (250–300 ng/mg), tubular degeneration, and a marked upregulation of injury biomarkers Kim‐1 and Ngal—the administration of α‐HgS resulted in negligible renal mercury levels (2–3 ng/mg), an absence of histopathological lesions, and no significant alteration of these sensitive molecular indicators of injury, thereby confirming its distinct and favorable safety profile [22, 30]. Therefore, the convergence of evidence from chronic in vivo studies, advanced materials science, and molecular‐to‐organismal functional assays provides a robust and multifaceted scientific foundation for the safety of LC, firmly establishing that its nanoengineered HgS core constitutes a pharmacologically distinct entity with a well‐defined and favorable toxicological profile.

### 5.2. A Meta‐Therapeutic Framework: Synergistic Components of a Self‐Regulating System

The clinical success and safety observed here are hypothesized to stem from the emergent properties of a rationally designed polypharmacological system constructed primary by five components. This system is architected to simultaneously leverage the immunomodulatory potential of nano‐HgS while embedding multiple layers of bioresponse modulation to ensure safety and efficacy (Table 6).

**TABLE 6:** The self‐regulating, integrative meta‐therapeutic system: Molecular targets, safety mechanisms, and emergent synergies.

System component	Proposed molecular targets and mechanisms in PG pathophysiology	Intrinsic safety and bioresponse modulation	Role in system synergy and emergent self‐regulation
1. HgS nanoparticles (Linga Chenduram)	Core immunomodulator: functions as a network stabilizer, not a blunt immunosuppressant. Proposed to modulate the ubiquitin–proteasome system via interaction with S‐phase kinase‐associated protein (Skp1), potentially delaying IκB degradation and thereby restraining the master inflammatory regulator NF‐κB [31, 32]. This is evidenced by the inhibition of P105 phosphorylation, leading to broad‐spectrum downregulation of NF‐κB‐driven cytokines (IL‐1β, IL‐6, IL‐8, TNF‐α) and chemokines central to PG’s cytokine storm and neutrophilic infiltrate [33, 34].	Fundamental safety by design: Its bioreactivity is fundamentally distinct from toxic mercurials. The traditional synthesis yields a stable, nanocrystalline α‐HgS phase with a robust covalent lattice, presenting a high kinetic barrier to dissociation. This results in extremely low solubility and ionic bioavailability, preventing the release of bioactive Hg^2+^ ions and the consequent oxidative stress and protein dysfunction that define mercury toxicity [9, 10, 18, 27].	Provides the primary, multitarget immunomodulatory signal. Its proposed NF‐κB inhibition creates a foundational “calming” of the immune network, which is amplified and safeguarded by all other components. Its safety profile is not inherent but is enabled and enforced by the synergistic system.

2. *Allium sativum*	Multitarget anti‐inflammatory and tissue stabilizer: exerts broad‐spectrum effects by suppressing NF‐κB and directly inhibiting key PG cytokines (IL‐1β, IL‐6, IL‐8, TNF‐α) and C‐reactive protein [35–38]. Critically, it inhibits pathological angiogenesis by reducing VEGF expression and stabilizes the extracellular matrix by downregulating MMP‐2 and MMP‐9, directly countering the tissue destruction in PG ulcers [35, 36].	Active mercury detoxification and cellular Shield: serves as a primary bioresponse modulator. Its sulfur compounds directly chelate mercury, forming less toxic sulfur adducts (e.g., bismethylmercury sulfide), reducing ionic bioavailability and tissue accumulation [39, 40]. Concurrently, it provides potent antioxidant and antiapoptotic protection, attenuating oxidative stress and suppressing caspase‐3–mediated apoptosis in organs like the liver and brain, thereby nullifying potential mercury toxicity [40–45].	Creates a powerful positive feedback loop: It directly augments the anti‐inflammatory and tissue‐stabilizing goals of the system while simultaneously creating a protective physiological milieu that ensures the safe application of the nano‐HgS core. It is both a therapeutic synergist and an essential safety buffer.

3. *Aloe vera*	Complementary pathway inhibition wound microenvironment modulator: Provides synergistic anti‐inflammatory by concurrently inhibiting the NF‐κB, JNK, and ERK signaling pathways, reducing the production of IL‐6, TNF‐α, and other mediators [46, 47]. Its inhibition of MMP‐9 is particularly critical for stabilizing the PG wound bed and facilitating re‐epithelialization, complementing garlic’s action on MMPs [46, 47].	Antioxidant defense system: Reinforces the system’s safety through its portfolio of polyphenolic antioxidants (e.g., aloe emodin). These compounds act as potent reactive oxygen species (ROS) scavengers and inhibitors of lipid peroxidation, mitigating a primary mechanism of heavy metal toxicity and further protecting cellular integrity [48].	Offers parallel pathway inhibition to garlic, ensuring a more robust and resilient suppression of the inflammatory network. Its antioxidant shield works in concert with garlic’s detoxifying mechanisms, forming a comprehensive safety net that manages the bioreactivity of the HgS nanoparticles.

4. Low‐Dose Prednisolone	Acute‐phase immunological Stabilizer: imposes immediate, broad‐spectrum anti‐inflammatory control via genomic mechanisms. Binds the glucocorticoid receptor to transrepress proinflammatory transcription factors (NF‐κB, AP‐1) and transactivate anti‐inflammatory genes (e.g., IκBα, GILZ), rapidly suppressing cytokine synthesis and immune cell migration [48].	Risk mitigation buffer: functions as an immunological “circuit breaker” during the acute treatment phase. Its powerful anti‐inflammatory action theoretically buffers against any transient, unforeseen proinflammatory fluctuations that could be triggered by the nascent interaction of HgS nanoparticles with a highly dysregulated immune system, a risk underscored by the known inflammatory potential of other mercury species [14–16, 49].	Provides temporal synergy by stabilizing the immune system at the initiation of therapy, creating a permissive window for the slower‐acting, recalibrating effects of the nano‐HgS and botanicals to take hold. It prevents acute exacerbations, thereby ensuring system stability and patient safety during the critical initial phase.

5. Optimized Physiological Milieu	Systemic foundation for biorecognition: The anti‐inflammatory, phytonutrient‐rich, low‐sodium diet directly quells systemic inflammation by suppressing NF‐κB and upregulating Nrf2‐mediated antioxidant defenses. Sodium restriction specifically inhibits the polarization of proinflammatory Th17 cells, a key pathway in autoimmunity [49–52].	Reduction of allostatic load: The controlled environment (pollution‐free, stress‐minimized, optimized sleep) systemically lowers the oxidative and inflammatory burden. This reduces the “noise” and hyper‐reactivity of the immune system, thereby raising the threshold for aberrant immune activation and favoring therapeutic over pathogenic biorecognition of the intervention [50–53].	Provides the foundational, system‐wide context for all other components to function effectively. By reducing the overall immune burden, it enhances the signal‐to‐noise ratio of the active therapeutics, making the immune system more receptive to recalibration. It is the essential canvas upon which the precise pharmacological picture is painted.

#### 5.2.1. Nano‐HgS as the Central Node in a Self‐Regulated Integrated Multitarget Therapeutic System

The notable clinical improvement observed in our PG cases, particularly the simultaneous resolution of cutaneous and articular inflammation, necessitates a hypothesis for the mechanistic role of the nanoengineered HgS in LC. While the exact molecular pathway in human PG requires further elucidation, the clinical outcome, combined with emerging research on nano‐HgS, suggests a multifaceted engagement with PG’s dysregulated immune network. We propose that LC does not function as a simple immunosuppressant, but as a modulator of protein stability and inflammatory signaling, potentially normalizing the exaggerated immune response characteristic of PG.

The primary immunomodulatory action may originate at the post‐translational level. Recent research identifies S‐phase kinase‐associated protein (Skp1) as a specific target of nano‐HgS [31]. As a core component of the Skp1‐cullin‐F‐box (SCF) ubiquitin ligase complex, Skp1 is essential for the targeted degradation of IκB, the endogenous inhibitor of NF‐κB [32]. By interacting with Skp1, nano‐HgS could potentially modulate the ubiquitin–proteasome system, delaying the degradation of IκB and thereby restraining the aberrant activation of the NF‐κB pathway [31, 32]. This is of paramount relevance in PG, where NF‐κB is a master regulator of the proinflammatory gene program, driving the expression of IL‐1β, IL‐6, IL‐8, TNF‐α, and other cytokines central to the disease’s pathophysiology [33]. This proposed mechanism is strongly supported by in vivo evidence demonstrating that HgS nanoparticle‐loaded hydrogels significantly reduce inflammation in rodent models by specifically inhibiting the phosphorylation of P105, a precursor to the NF‐κB p50 subunit [34]. The consequent downregulation of this central signaling hub would logically lead to a broad‐spectrum reduction in the cytokine storm that propagates tissue damage in PG.

Furthermore, the impact on NF‐κB may extend to its interplay with other dysregulated pathways in PG. The JAK‐STAT signaling pathway, frequently hyperactive in PG, can be influenced by NF‐κB activity and vice versa [3, 33]. By dampening the primary NF‐κB signal, LC may indirectly contribute to a broader normalization of immune crosstalk. This system‐level effect aligns with the clinical observation of concurrent improvement in both skin lesions and seronegative arthropathy, conditions that share underlying inflammatory mechanisms. Therefore, the probable mechanism of LC in PG involves a foundational regulation of the NF‐κB pathway, potentially via Skp1 interaction, leading to a downstream cascade of reduced cytokine production, neutrophil chemotaxis, and tissue inflammation, ultimately facilitating the observed wound healing and restoration of immune homeostasis. This hypothesis provides a rigorous scientific framework for the notable clinical outcomes and directs future research to validate these specific molecular interactions in the context of PG.

#### 5.2.2. *Al. sativum* and *A. vera* as Phytomedical Synergists: Orchestrating Multitarget Anti‐Inflammation and Mercury Bioresponse Modulators of This Self‐Regulating, Integrative Meta‐Therapeutic System

Within this integrative meta‐therapeutic system, *Al. sativum* and *A. vera* function as indispensable phytomedical synergists, orchestrating a dual, self‐regulating role that simultaneously dismantles the pathogenic complexity of PG and intrinsically modulates the bioreactivity of its mercury‐based core. Their primary therapeutic action is a sophisticated, multitargeted assault on the hyperinflammatory and tissue‐destructive pathways central to PG. The molecular efficacy of *Al. sativum* is profoundly rooted in its rich repertoire of organosulfur compounds, which potently suppress the master inflammatory regulator NF‐κB, leading to a concurrent downregulation of the key cytokines that fuel the PG‐associated “cytokine storm” including IL‐1β, IL‐6, IL‐8, and TNF‐α [35, 36]. This broad‐spectrum cytokine inhibition is strategically complemented by a direct impact on tissue remodeling and angiogenesis; garlic stem extract demonstrably inhibits the gene expression of matrix metalloproteinase (MMP)‐2 and MMP‐9, while its constituents also suppress vascular endothelial growth factor (VEGF), thereby curbing the pathological angiogenesis and proteolytic tissue degradation that characterize nonhealing ulcers [35, 36]. The systemic validity of this anti‐inflammatory prowess is confirmed by meta‐analyses, showing that garlic supplementation significantly reduces circulating levels of CRP, TNF‐α, and IL‐6 [37]. In a powerful synergistic parallel, *A. vera* extracts enact a complementary immunomodulatory strategy by concurrently targeting the NF‐κB and MAPK (JNK and ERK) signaling cascades, thereby inhibiting the downstream production of pivotal mediators such as IL‐6, TNF‐α, and critically, MMP‐9 [46, 47]. This multipronged phytopharmacological attack directly counteracts the PG pathophenotype, where the overexpression of MMP‐9 and MMP‐10, coupled with a deficit in epithelial MMPs, creates a microenvironment of relentless tissue destruction [1–3, 54]. Furthermore, the specific inhibition of VEGF by garlic directly addresses the dysregulated angiogenic processes implicated in PG pathogenesis, which are driven by factors like VEGF and hypoxia‐inducible factor‐2 [55], thereby helping to normalize the vascular bed and facilitate structured wound healing. Beyond this direct anti‐inflammatory orchestration, the second and fundamentally critical role of these botanicals is to function as intrinsic bioresponse modulators for the HgS nanocore. *Al. sativum* employs a dual mechanism for mercury mitigation: Its lipophilic sulfur compounds directly chelate mercury to form less toxic sulfur adducts like bismethylmercury sulfide, thereby reducing its biological reactivity and bioavailability [39], while its potent antioxidant and antiapoptotic constituents actively protect against mercury‐induced cellular damage by attenuating oxidative stress, inhibiting caspase‐3 activation, and preventing apoptotic cell death in critical organs like the brain and liver [40, 41]. This is corroborated by in vivo evidence demonstrating that garlic coadministration significantly reduces mercury accumulation in tissues and protects against hepatotoxicity and neurotoxicity [42–45]. *A. vera* reinforces this protective shield through its portfolio of polyphenolic antioxidants, such as aloe emodin, which function as potent scavengers of reactive oxygen species and inhibitors of lipid peroxidation, thereby mitigating a primary mechanism of heavy metal toxicity [38]. Collectively, this sophisticated phytomedical synergy creates a self‐regulating therapeutic system where garlic and *A. vera* not only directly deconstruct the immunopathogenic network of PG through cytokine, MMP, and VEGF inhibition but also establish a safeguarded physiological milieu that shapes the bioreactivity of the nano‐HgS. This ensures that the potent, immunomodulatory signal of nano‐HgS can be delivered without the attendant risks of mercury toxicity, thereby enabling the entire meta‐therapeutic system to effectively recalibrate immune dyshomeostasis in refractory PG, a proposed mechanism that aligns with the profound clinical responses observed.

#### 5.2.3. Low‐Dose Prednisolone: An Acute‐Phase Immunological Stabilizer

The short‐course, low‐dose prednisolone functions as a critical acute‐phase immunological stabilizer. Its role is to impose immediate, broad‐spectrum anti‐inflammatory control, rapidly mitigating the pathogenic cytokine storm in PG by leveraging the core molecular mechanisms of glucocorticoid action. Prednisolone binds to the cytosolic glucocorticoid receptor (GR), leading to its nuclear translocation where it orchestrates a dual genomic strategy: It transrepresses proinflammatory transcription factors like NF‐κB and AP‐1, thereby directly suppressing the synthesis of cytokines (e.g., IL‐1, IL‐6, TNF‐α), and simultaneously transactivates genes encoding anti‐inflammatory proteins such as IκBα and GILZ [48]. This transcriptional reprogramming stabilizes the immune milieu, providing immediate symptom control and, crucially, creating a permissive environment for the nano‐HgS to exert its immunomodulatory effects. This buffering action is essential for risk mitigation, as it theoretically prevents any transient proinflammatory fluctuations that could be triggered by the nascent interaction of nano‐HgS with a dysregulated immune system, a safeguard supported by evidence that mercury species can provoke inflammatory and autoimmune responses [14–16].

#### 5.2.4. Optimized Physiological Milieu: The Systemic Foundation for Therapeutic Biorecognition

The anti‐inflammatory, antioxidant‐rich diet, and stress‐minimized inpatient setting are active therapeutic components that establish a critical systemic foundation. This controlled environment functions by reducing the systemic oxidative and inflammatory burden, thereby lowering the threshold for aberrant immune activation and creating an optimized physiological milieu that favors the therapeutic over the potential pathogenic bioreactivity of the intervention [49]. Molecularly, the phytonutrient‐rich, low‐sodium diet directly suppresses proinflammatory pathways, including NF‐κB, while upregulating endogenous antioxidant defenses such as Nrf2, thereby systemically quelling the inflammatory background that fuels autoimmunity [49–51]. Concurrently, sodium restriction has been demonstrated to modulate immunoregulation by inhibiting the polarization of proinflammatory Th17 cells, a lymphocyte subset critically implicated in the pathogenesis of PG and other autoimmune conditions [52]. This nutritional strategy possesses a dual function: It not only directly counteracts the disease pathophysiology but also proactively shapes the bioreactivity of the integrated therapeutics. The provision of antioxidant‐rich fruits and vegetables may further support the mercury‐chelating capacity of the protocol, as certain phytonutrients can facilitate the reduction and mobilization of heavy metals [53]. By synergistically combining this dietary regimen with a controlled environment that minimizes physical and mental stressors, the protocol effectively reduces allostatic load. This comprehensive approach establishes a permissive systemic state that enhances the biorecognition and efficacy of the core immunomodulatory agents—nano‐HgS, garlic, and *A. vera*—while mitigating potential adverse reactions, thereby enabling a more precise and successful recalibration of the dysregulated immune response in refractory PG.

### 5.3. Justifying the Core Role of Nano‐HgS: Orchestrating Immune Recalibration Beyond Conventional Therapy

The compelling clinical outcomes observed—rapid ulcer re‐epithelialization, resolution of systemic inflammation, and sustained remission—cannot be adequately explained by the action of low‐dose prednisolone and botanicals alone. While *Al. sativum* and *A. vera* provide robust, multitarget anti‐inflammatory and tissue‐stabilizing effects, their pharmacodynamics are inherently limited to the modulation of known inflammatory pathways (e.g., NF‐κB, MAPK, cytokine production) [35–37, 46, 47]. In refractory PG, characterized by a deeply entrenched, self‐amplifying immune dysregulation [1–3], this phytopharmacological intervention often proves insufficient, as evidenced by the failure of prior conventional immunosuppressants. The nano‐HgS in LC is proposed to function not merely as an additive anti‐inflammatory agent but as the core architectural component that transforms the system from a combination therapy into an emergent, self‐regulating therapeutic entity.

The necessity of nano‐HgS stems from its hypothesized role as a master regulator of protein homeostasis and immune signal persistence. The proposed interaction with Skp1 and the ubiquitin–proteasome system represents a fundamentally different level of intervention [31]. By potentially modulating the degradation rate of IκB and other key regulatory proteins, nano‐HgS does not just inhibit the NF‐κB pathway transiently; it may reprogram the intrinsic feedback loops and activation thresholds of the immune system. This action is akin to “immune response recalibration” [56], resetting a misaligned circuit board, whereas cytokine inhibitors merely mute the output of a single speaker. This proposed mechanism aligns with the clinical observation of sustained remission long after the short‐course steroids were discontinued, suggesting that a durable recalibration of immune homeostasis was achieved.

Furthermore, the nano‐HgS core is postulated to be the key driver of therapeutic synergy within the system. Its presence as an inorganic nanomaterial creates a unique biointerface that alters the pharmacological context for the other components, a role that organic compounds alone cannot fulfill, aligning with the broader paradigm of nanotechnology‐mediated immunomodulation [57]. The botanicals (*Al. sativum*, *A. vera*), beyond their direct anti‐inflammatory and tissue‐healing roles, are not merely adjuvants but essential bioresponse modulators that shape the interaction between the nano‐HgS and the host’s biology. They create a safeguarded milieu that permits the potent, network‐level signal of the nano‐HgS to be delivered without triggering the proinflammatory or toxic responses characteristic of uncontrolled mercury exposure, a critical consideration given that the immune response to inorganic nanoparticles and their degradation products is distinct from their ionic or bulk forms [58]. The low‐dose prednisolone acts as an acute‐phase stabilizer, buffering the system during initial engagement. The optimized physiological milieu provides the foundational context that reduces systemic “noise,” allowing for clearer biorecognition of the therapeutic signal.

Therefore, removing the nano‐HgS from this system would be analogous to removing the conductor from an orchestra; the individual musicians (botanicals, steroids) can play their parts, but the complex, synchronized, and transformative performance—the emergent self‐regulation required to manage refractory PG—fails to materialize. The nano‐HgS is hypothesized to be the indispensable element that confers upon the integrated system its potent steroid‐sparing capacity and its ability to achieve definitive healing in otherwise recalcitrant disease.

### 5.4. Emergent Polypharmacology: From Molecular Targeting to System‐Level Resolution

The consistent multisystem remission observed across our cohort—encompassing both refractory cutaneous ulceration and associated seronegative arthropathy—validates the central thesis of our meta‐therapeutic strategy: It successfully engages the shared immunological axis of pan‐autoinflammation. Unlike single‐target biologics that selectively mute individual cytokine signals [6, 7], our integrated system demonstrates orchestrated modulation across the dysregulated network, simultaneously suppressing pivotal cytokines (IL‐1β, IL‐6, IL‐8, IL‐17, TNF‐α), inhibiting tissue‐destructive metalloproteinases, and dampening the hyperactive NF‐κB and JAK‐STAT signaling cascades. This emergent polypharmacology is the functional manifestation of the system’s self‐regulating architecture, where the foundational signal of the nano‐HgS core is amplified and contextualized by the botanicals’ pathway modulation and the steroid’s acute‐phase stabilization. Consequently, this approach transcends the limitations of conventional immunosuppression, not by being more potent in a single pathway, but by being more intelligent across the entire network. It thereby establishes a new therapeutic archetype for recalcitrant autoinflammatory disease: a steroid‐sparing modality that achieves resolution by restoring dynamic equilibrium, not through maximal suppression.

### 5.5. Limitations and the Imperative for Mechanistic Deconstruction

While the clinical outcomes presented are notable, this hypothesis‐generating case series is intrinsically limited by its observational design, small cohort size, and the absence of a control group, which precludes definitive causal attribution of the observed healing to the intervention. Furthermore, while long‐term safety biomonitoring at 6 months revealed negligible mercury levels (Table 5), the absence of serial pharmacokinetic data—particularly immediate pre‐ and postintervention measurements—limits our understanding of the absorption, peak exposure, and clearance dynamics of the nano‐HgS core during the active treatment period. The proposed molecular mechanisms, while grounded in extrapolation from disparate research models, remain speculative; the true in vivo pharmacodynamic interactions within this complex system in human PG are unknown and likely involve sophisticated, unelucidated biological networks beyond our current hypotheses. Crucially, the well‐established and severe toxicology of most mercury species mandates that its therapeutic application not be justified by clinical tradition alone but be radically reframed through the rigorous lens of modern nanoscience, toxicology, and systems biology. This study, therefore, does not present a concluded therapeutic formula but rather a provocative pilot dataset that necessitates a fundamental shift from historical anecdote to a molecular‐level, evidence‐based understanding of this potent material’s therapeutic potential and risks.

## 6. Future Directions: A Translational Roadmap for Next‐Generation Meta‐Therapeutics

To address these limitations and responsibly advance this field, a concerted, multipronged research agenda is imperative. Future efforts must be stratified across several domains:

### 6.1. Mechanistic Deconstruction

The highest priority is the de novo molecular characterization of nano‐HgS’s action. This requires advanced in vitro and in vivo models of neutrophilic inflammation, utilizing transcriptomics, proteomics, and metabolomics to map the global immune recalibration induced by the full meta‐therapeutic system versus its individual components. This will identify the unique molecular signature of the nano‐HgS core and validate its proposed role as a Skp1/ubiquitin–proteasome modulator.

### 6.2. Clinical Validation and Standardization

A large‐scale, randomized controlled trial (RCT) against the current standard of care (e.g., systemic corticosteroids or cyclosporine) is essential to establish efficacy and safety rigorously. This must be coupled with stringent heavy metal biomonitoring protocols to define the compound’s pharmacokinetic profile and establish clear safety parameters.

### 6.3. Pharmaceutical Innovation

The principles uncovered should guide the rational design of next‐generation, synthetic nanoimmunomodulators inspired by the nano‐HgS architecture but engineered for enhanced biocompatibility and a more predictable therapeutic index, potentially utilizing less toxic metal cores or fully organic nanomimetics.

### 6.4. Ethical and Regulatory Framework

The provocative findings herein underscore the urgent need for international guidelines governing the research and clinical use of refined herbo‐metallic compounds. This includes strict protocols to prevent misuse, a clear demarcation between pharmaceutically processed nanoforms and toxic environmental mercury, and public health initiatives to eradicate uncontrolled mercury exposure.

### 6.5. Paradigm Shift in Therapeutic Design

Ultimately, this work advocates for a broader transition from a disease‐centric to a patient‐centric, mechanism‐based meta‐therapeutic approach. By leveraging multiomics biomarkers to deconstruct complex autoimmune pathologies, we can move toward constructing personalized, evidence‐based integrative systems capable of restoring network‐level homeostasis in refractory diseases, pointing toward a new direction in therapeutic design.

## 7. Conclusion

This study presents a transformative approach to the formidable challenge of refractory PG through a self‐regulating, integrative meta‐therapeutic system. The notable clinical outcomes—robust wound healing, resolution of systemic inflammation, and sustained remission—provide compelling preliminary evidence that a system‐level intervention, architected around a nanoengineered HgS core and synergistic phytocompounds, can achieve what single‐pathway agents often cannot: durable immune recalibration. We posit that the nano‐HgS functions as the indispensable conductor of this therapeutic orchestra, proposed modulating fundamental protein homeostasis to reset dysregulated immune networks. While the historical toxicity of mercury demands extreme caution and rigorous scientific validation, our findings suggest a nuanced reappraisal, distinguishing the uncontrolled exposure to toxic mercury species from the controlled application of a pharmaceutically refined, nanostructured entity within a safeguarded therapeutic context. This work does not offer a final solution but opens a new avenue for investigation, challenging the medical community to explore the sophisticated interface of traditional nanomedicine and modern immunology to develop a new class of powerful, steroid‐sparing therapies for recalcitrant autoimmune and inflammatory diseases.

## Funding

No funding was received for this manuscript.

## Ethics Statement

All investigations were conducted in accordance with the ethical principles of the Declaration of Helsinki. The study protocol was reviewed and approved by the Institutional Ethics Committee of MGACHRC. Prior to inclusion, a comprehensive informed consent process was undertaken for all participants. This involved a detailed explanation of the entire treatment protocol, with particular emphasis on the novel nature of the herbo‐metallic intervention and an explicit discussion of the potential risks associated with the use of a mercury‐based compound, including the known toxicological profile of mercury and the safety measures in place. Written informed consent for participation in the therapeutic protocol was obtained following this discussion.

Furthermore, separate, specific written consent was obtained from each patient for the collection, analysis, and publication of their anonymized medical data, personal clinical details, and clinical photographs. All treatments were administered in a controlled inpatient setting under strict supervision and according to a predefined protocol to ensure patient safety.

## Conflicts of Interest

The authors declare no conflicts of interest.

## Patient Perspective

All four patients reported profound satisfaction with their treatment outcomes, emphasizing not only the dramatic physical healing of their ulcers but also a transformative restoration of functional mobility and psychological well‐being. They expressed that the comprehensive nature of the care—encompassing diet, environment, and medication—was instrumental in their recovery, providing a sense of control and active participation in the healing process that had been absent in their prior, fragmented treatment experiences.

## Data Availability

The data that support the findings of this study are not publicly available but can be obtained from the corresponding author upon reasonable request.
